# Daily supplementation of a multiple micronutrient powder improves folate but not thiamine, riboflavin, or vitamin B_12_ status among young Laotian children: a randomized controlled trial

**DOI:** 10.1007/s00394-022-02890-3

**Published:** 2022-05-09

**Authors:** Guy-Marino Hinnouho, Daniela Hampel, Setareh Shahab-Ferdows, Maxwell A. Barffour, Liadhan McAnena, Charles D. Arnold, K. Ryan Wessells, Sengchanh Kounnavong, Lindsay H. Allen, Helene McNulty, Sonja Y. Hess

**Affiliations:** 1 Department of Nutrition, Institute for Global Nutrition, University of California, Davis, CA USA; 2 Helen Keller International, Washington, DC USA; 3 USDA, ARS Western Human Nutrition Research Center, Davis, CA USA; 4 University of Missouri School of Medicine, Columbia, MO USA; 5 Public Health Program, College of Health and Human Services, Missouri State University, Springfield, MO USA; 6 Nutrition Innovation Centre for Food and Health (NICHE), School of Biomedical Sciences, Ulster University, Coleraine, Northern Ireland UK; 7 Lao Tropical and Public Health Institute, Vientiane, Lao People’s Democratic Republic

**Keywords:** Micronutrient powder, MNP, Thiamine, Riboflavin, Folate, Vitamin B_12_, Young children, Lao PDR

## Abstract

**Purpose:**

To assess the effects of intervention with a daily multiple micronutrient powder (MNP) on thiamine, riboflavin, folate, and B_12_ status among young Laotian children.

**Methods:**

Children (*n* = 1704) aged 6–23 mo, participating in a double-blind placebo-controlled randomized trial were individually randomized to receive daily either MNP (containing 0.5 mg of thiamine, 0.5 mg riboflavin, 150 μg folic acid, and 0.9 μg vitamin B_12_ along with 11 other micronutrients) or placebo and followed for ~ 36 weeks. In a randomly selected sub-sample of 260 children, erythrocyte thiamine diphosphate (eThDP), plasma folate and B_12_ concentrations, and erythrocyte glutathione reductase activation coefficient (EGRac; riboflavin biomarker) were assessed at baseline and endline.

**Results:**

There was no treatment effect on endline eThDP concentrations (110.6 ± 8.9 nmol/L in MNP vs. 109.4 ± 8.9 nmol/L in placebo group; *p* = 0.924), EGRac (1.46 ± 0.3 vs. 1.49 ± 0.3; *p* = 0.184) and B_12_ concentrations (523.3 ± 24.6 pmol/L vs. 515.9 ± 24.8 pmol/L; *p* = 0.678). Likewise, the prevalence of thiamine, riboflavin, and B_12_ deficiencies did not differ significantly between the two groups. However, endline folate concentration was significantly higher in the MNP compared to the placebo group (28.2 ± 0.8 nmol/L vs 19.9 ± 0.8 nmol/L, respectively; *p* < 0.001), and correspondingly, the prevalence of folate deficiency was significantly lower in the MNP group (1.6% vs 17.4%; *p* = 0.015).

**Conclusions:**

Compared to a placebo, daily MNP for 9 months increased only folate but not thiamine, riboflavin, or B_12_ status in young Laotian children.

**Trial registration:**

The trial was registered at www.clinicaltrials.gov (NCT02428647) on April 29 2015.

## Introduction

Micronutrient deficiencies lead to growth and mental impairments, cognitive delays, and weakened immunity, and contribute to childhood morbidity and mortality [1]. While deficiencies in iron, zinc, iodine, and vitamin A are recognized as major public health concerns among young children and women of reproductive age [1, 2], deficiencies in micronutrients such as thiamine, riboflavin, folate, and vitamin B_12_ are also increasingly being reported [3–6].

B-vitamins regulate key physiological functions in the body [7]. Thiamine regulates glucose metabolism, and plays a key role in brain development and neuronal activity in children [7, 8]. Riboflavin is an essential coenzyme for redox reactions in many different metabolic pathways. It is the precursor of flavin adenine mononucleotide and flavin adenine dinucleotide which play an important role in amino-acid and energy metabolism [7]. Riboflavin is also involved in the metabolism of vitamin B_6_, folate, and B_12_ [9]. Folate and B_12_ are crucial for brain development and function, and are important for mental and emotional health during infancy and early childhood [10]. These latter two vitamins are also closely involved in red blood cell metabolism.

Deficiencies of these vitamins can have devastating consequences, especially in children. Thiamine deficiency occurs mainly in breastfed infants of mothers who have inadequate intake of thiamine and clinically apparent thiamine deficiency, also known as beriberi, may lead to cardiac insufficiency, peripheral neuropathy, encephalopathy, and even death [11–14]. Riboflavin deficiency is usually due to dietary inadequacy (milk, meat, dairy products, and eggs) and is associated with peripheral neuropathy, impaired iron absorption, and poor growth in children [15]. Folate and B_12_ deficiencies occur mainly in infants born from folate and B_12_-deficient mothers in populations with poor or inadequate dietary intake of leafy vegetables and meat [16] and deficiency in either vitamin can affect growth in young children [17]. Folate and to some extent B_12_ deficiencies cause megaloblastic anemia, low birthweight, and neural tube defects [18]. A typical meal in Laos consists of rice complemented with small portions of vegetables, mainly green leafy vegetables and fish. Other common food items are roots, eggs, meat, poultry, and various kinds of fruit. Nevertheless, according to the Lao Social Indicator Survey [19], only 27, 36, and 43% of children aged 6–23 months met minimum dietary scores indicating minimum acceptable diet, minimum dietary diversity, and minimum meal frequency, respectively.

Thiamine deficiency is increasingly being recognized as a public health problem in Southeast Asia [5, 13, 20]; in Vientiane, Lao PDR, a recent study of infants without clinical signs of thiamine deficiency admitted to a hospital over the course of a year, found that 13.4% were thiamine deficient [21]. Another study in northern Lao PDR reported that 30% of Laotian children were thiamine deficient and suggested that beriberi may be a major cause of infant mortality [22]. While there is an extensive literature on riboflavin status among school-age children and adult populations in both high-income and low- and middle-income countries [23–26], recent reports of biochemical and clinical data for riboflavin status and prevalence of riboflavin deficiency in children under 5 years are scarce. Recent trials reported a prevalence of riboflavin deficiency of 3.3% in Peru [27] and 22.4% in South Africa among 6–12 month old children [28]. Previous studies in India and Nepal have reported a high prevalence of B_12_ deficiency in young children (30–36%) but low (2–3.2%) or no prevalence of folate deficiency [29–32]. Whether deficiencies of riboflavin, folate, and B_12_ are common in young Laotian children is presently unknown.

Children who are deficient in one micronutrient are often at risk for other deficiencies [33]; thus, supplementation with multiple micronutrients (MMN) such as lipid-based nutrient supplements (LNS) and micronutrient powders (MNP) may be more beneficial than supplementation with a single micronutrient. While studies on thiamine-, folic acid-, or B_12_- supplementation of pregnant and lactating women have reported an improvement in the breastfed infants’ status of these micronutrients [15, 34–37], results from interventions such as MMN supplements and LNS among young children have been inconclusive with respect to riboflavin, folic acid, and B_12_ status [27, 28, 38–42]. Worldwide, a considerable number of MNP interventions have been conducted in different settings, and their efficacy in the prevention of iron deficiency and anemia has been demonstrated [43, 44]. However, there is a lack of evidence on the effect of MNP supplementation on B-vitamin status in young children.

In the present study, we assessed the impact of an MNP (containing 0.5 mg of thiamine, 0.5 mg of riboflavin, 150 μg of folic acid, and 0.9 μg of vitamin B_12_ along with 11 other micronutrients) on thiamine, riboflavin, folate, and B_12_ status of young children participating in a randomized controlled trial in Lao PDR. We hypothesized that children who are supplemented daily with MNP for 36 weeks will have higher concentrations of thiamine, riboflavin, folate, and B_12_ compared to children in the control group. In addition, we evaluated whether deficiencies of thiamine, riboflavin, folate, and B_12_ are common in this population and examined the risk factors associated with these deficiencies at baseline.

## Methods

### Study design, participants, and randomization

The Lao Zinc Study was a randomized controlled double-blind community-based trial implemented from September 2015 until April 2017 in rural communities of Khammouane Province in Central Lao PDR. This area was chosen because of a high prevalence of stunting and underweight of young children (~ 41% stunting prevalence and ~ 29% underweight prevalence among under-five children) [19] and the lack of programs implemented at the time to reduce the risk of micronutrient deficiencies. The study protocol and the consent procedure were approved by the National Ethics Committee for Health Research (Lao PDR) and the Institutional Review Board of University of California, Davis, USA. The trial was registered at www.clinicaltrials.gov (NCT02428647).

The primary objective of the Lao Zinc Study was to compare the effects of two forms of daily preventive zinc supplementation (tablets and MNP) versus therapeutic zinc supplementation for diarrhea on young children’s physical growth and other health outcomes. A detailed protocol of the Lao Zinc Study has been published elsewhere [45]. Briefly, written informed consent (documented by either a signature or a fingerprint) was obtained from one of the child’s primary caregivers (mother, father, or legal guardian) in the presence of an impartial witness. Children were considered eligible if they were 6–23 months of age, and their families accepted weekly in-home visits, planned residency within the study area for the duration of the study, and provided informed consent. Children were ineligible if they met one of the following criteria: severe anemia (Hb < 70 g/L), weight-for-length *z *score (WLZ) <  − 3 SD [46], presence of bipedal edema, severe illness warranting hospital referral, congenital abnormalities potentially interfering with growth, chronic medical condition (e.g., malignancy) requiring frequent medical attention, known human immunodeficiency virus (HIV) infection of index child or child’s mother, currently consuming zinc supplements or current participation in another clinical trial.

A statistician at University of California Davis randomly assigned the study ID numbers to the 4 study arms, using a block randomization scheme with block lengths of 4 or 8. A total of 3407 children 6–23 mo of age were enrolled in the main trial and individually randomized to one of four intervention groups: (1) the preventive zinc supplementation group, who received a daily preventive zinc supplement tablet containing 7 mg of zinc and placebo therapeutic tablets for diarrhea; (2) the micronutrient powder group (MNP), who received a daily preventive micronutrient powder containing 10 mg of zinc, 6 mg of iron, and 13 other micronutrients and placebo therapeutic tablets for diarrhea; (3) the therapeutic zinc supplementation group, who received a daily placebo preventive supplement tablet and therapeutic zinc tablets containing 20 mg for 10 days for diarrhea treatment; or (4) the placebo control group, who received daily placebo preventive powder and placebo therapeutic tablets for diarrhea. In all groups, children remained under observation and received their assigned supplements for a period of 36 weeks. For the present analyses, only children randomized to the MNP or the placebo control groups (*n* = 1704) were considered.

### Intervention products and supplement administration

The MNP and placebo powder supplements were produced by DSM Fortitech Asia Pacific Sdn Bhd (Banting, Malaysia). One MNP sachet provided the following micronutrients daily: 400 µg RAE vitamin A, 0.5 mg thiamine as thiamine mononitrate, 0.5 mg riboflavin as riboflavin, 6 mg niacin as niacinamide, 0.5 mg vitamin B_6_ as pyridoxine hydrochloride, 150 µg DFE folic acid, 0.9 µg cyanocobalamin, 30 mg ascorbic acid, 5 mg cholecalciferol, 5 mg TE dl-α-tocopheryl acetate, 0.56 mg copper as copper sulfate anhydrous, 90 µg iodine as potassium iodate, 6 mg iron as ferrous fumarate, 17 µg selenium as selenium selenite, and 10 mg zinc as zinc gluconate. Maltodextrin was included as an excipient in both the placebo and the MNP sachets. All intervention products were coded with a two-digit group code and a group-specific color. Caregivers were instructed to mix the entire content of the sachet with a semi-solid or mashed food. Caregivers were encouraged to mix the powder package into suitable foods, such as mashed mango, banana and papaya, boiled pumpkin, and boiled egg [47].

Only one child per household was eligible to participate in the study. In the event that there was more than one eligible child per household, only the youngest was enrolled. In the case of twins, both twins were assigned to the same group and received all study-related interventions and followed-up, but only one was randomly selected for inclusion in the data analyses. Each household was visited weekly by a morbidity surveillance worker who delivered the respective MNP or placebo for ~ 36 weeks. During the weekly visit, morbidity and adherence to supplementation were assessed based on caregiver’s report and collection of empty packages.

### Data collection

Children’s anthropometry [weight, length, and mid-upper arm circumference (MUAC)] were measured at baseline, mid-point (18 week follow-up), and endline, and maternal weight and height were measured once either at baseline, or at the next measurement round. All anthropometric measurements were completed in duplicate following protocols recommended by the Food and Nutrition Technical Assistance Project [48]. Information on maternal and household demographic and socio-economic status (education, occupation, ethnicity, household size and composition, housing material, household assets, and land ownership), household food security, and hygiene and sanitation practices were collected at baseline. Information on infant and young child feeding (IYCF) practices (breastfeeding, formula feeding, 24 h and 7 day food frequency questionnaire) were collected at baseline and every 4 weeks.

At baseline and endline assessments, venous blood samples from an antecubital, dorsal metacarpal, or great saphenous vein of children who were not acutely ill, were collected into evacuated, trace element-free 7.5 ml polyethylene blood collection tubes containing lithium heparin (Sarstedt AG & Co, Numbrecht, Germany; ref 01.1604.400) and stored at 4–8 °C until transportation to the project laboratory. The blood was centrifuged within ≤ 8 h of collection at 1097 × g (3100 RPM) for 10 min (PowerSpin Centrifuge Model LX C856; United Products & Instruments, Inc., Dayton, NJ) and plasma was aliquoted into pre-labeled microcentrifuge tubes, and stored at  − 20 °C. The buffy coat was carefully removed, and packed erythrocytes were washed three times in ~ 5 ml physiological saline (9 g/L NaCl). To hemolyze the cells, 0.5 mL distilled water was added to 0.5 mL aliquots of red blood cells (RBC) in 1.5 mL amber tubes, and vortexed for 1 min, and stored at  − 80 °C for the duration of the field study. Plasma and RBC samples were shipped on dry ice to the University of California, Davis and from there to collaborating laboratories.

### Laboratory analyses

Baseline and endline erythrocyte thiamine diphosphate (eThDP) and plasma folate and B_12_ concentrations were analyzed at the USDA/ARS Western Human Nutrition Research Center, Davis, CA, USA. The eThDP concentrations were determined using high-performance liquid chromatography with fluorescence detection (HPLC-FLD) of their thiochrome derivatives after pre-column derivatization [49]. Briefly, the analysis was carried out using an Agilent 1200 series HPLC System equipped with a fluorescence detector (l_ex:_ 367 nm, l_em_ 435 nm) and operated by ChemStation Rev. B.02.01.SR1 (Agilent Technologies, Santa Clara, CA). A pooled RBC sample from an apparently healthy donor was used as an internal control sample and prepared with every set of analyses (inter-assay variation of the pooled RBC sample for eThDP: 13.5%, *n* = 50).

The EGRac assay, providing a functional indicator of riboflavin status, is considered to be the gold standard method of assessment of biomarker status of riboflavin. In this method, the activity of the FAD-dependent enzyme, glutathione reductase, is measured in red blood cells before and after incubation with added FAD; the ratio of FAD-stimulated-to-unstimulated EGR activity thus indicates the degree of sample saturation with riboflavin. A higher EGRac ratio indicates poorer riboflavin status, with EGRac > 1.40 widely accepted as indicative of riboflavin deficiency. EGRac was performed at Ulster University, Coleraine, Northern Ireland, using established protocols on a Randox Daytona + clinical chemistry analyser (Randox Laboratories, Crumlin, Northern Ireland). Quality control was provided by repeated analysis of stored aliquots of pooled and characterized erythrocytes with known EGRac values corresponding to adequate and deficient status.

Plasma folate and B_12_ concentrations were assessed using the SimulTRAC-SNB Radioassay Vitamin B_12_ [^57^Co]/Folate [^125^I] Kit (MP Biomedicals). Inter-assay variation of the controls was 7.4–12.4% for folate and 0.3–0.4% for B_12,_ respectively.

Plasma samples were analyzed at the VitMin Lab (Willstaett, Germany) for biomarkers of iron (ferritin, soluble transferrin receptor (sTfR)), vitamin A [retinol-binding protein (RBP)], and inflammatory status [C-reactive protein (CRP) and α1-acid glycoprotein (AGP)] by combined sandwich enzyme-linked immunosorbent assay (ELISA) technique [50]

### Outcomes and definitions

The following primary outcomes were considered in the present study: (a) eThDP concentrations and prevalence of thiamine deficiency (eThDP < 70 nmol/L) [51]; (b) EGRac and prevalence of riboflavin deficiency (EGRac >  = 1.4) [51]; (c) folate concentrations and prevalence of folate deficiency (plasma folate < 10 nmol/L) [52]; (d) B_12_ concentrations and prevalence of B_12_ deficiency (plasma B12 < 221 pmol/L) [53].

Stunting, underweight, and wasting were defined as length-for-age *z* scores (LAZ) <  − 2 SD, weight-for-age *z* scores (WAZ) <  − 2 SD and weight-for-length *z* scores (WLZ) ) <  − 2 SD respectively [46]. Low MUAC was defined as MUAC ≤ 12.5 cm [54]. Elevated CRP and AGP were defined as CRP > 5 mg/L and AGP > 1 g/L respectively [55]. Low ferritin was defined as plasma ferritin (pF) < 12 µg/L [56] and high sTfR as sTfR > 8.3 mg/L [57]. Anemia was defined using the hemoglobin (Hb) cut-off for children 6–59 months of age (Hb < 110 g/L) [56]. Breastfeeding practices were assessed every 4 weeks over the course of the study, and a child was considered breastfed if breastfeeding was reported at least once in the past month.

### Sample size estimation

For the present study, a sample size of 130 children per intervention group (260 children in total) was estimated to be sufficient to detect an effect size of 0.35SD with 80% power and 5% of type I error using a two-sided test. Given the limited literature on the effect of micronutrient supplementation on these aforementioned B-vitamins, this sample size was informed by the estimated effect size of the intervention on other micronutrients such as plasma zinc and ferritin [58–60]. Thus, a random sub-sample of 260 children from children who had completed the study and with baseline and endline RBC and plasma samples (130 in the MNP group and 130 in the control group) was selected for the current analyses.

### Statistical analyses

A statistical analysis plan was developed and published before starting the analyses and unblinding the interventions group [61]. All analyses were carried out using Stata 14 for Windows (StataCorp. 2015, College Station, TX, USA). The primary outcomes were eThDP, EGRac, plasma folate, and B_12_ concentrations. The intervention group was considered the primary exposure variable and analyses were done following a complete-case intention-to-treat principle.

Principal components analysis was applied to available indicators of household socio-economic status, education, income, ownership of lands, and hygiene and sanitation practices to derive a socio-economic status (SES) index [62]. Food security was defined using the household food insecurity access scale (HFIAS) [63] and information on IYCF practices (breastfeeding, dietary diversity, and food frequency) was used to estimate minimum dietary diversity (MDD), minimum meal frequency (MMF), and consumption of iron-rich foods as specified by WHO [64, 64].

Treatment effects were assessed in both minimally adjusted models including baseline measurement of the outcome, child age at enrollment, and district of enrollment as well as adjusted models including variables in the minimally adjusted models and pre-specified variables determined to be associated with outcome (*p* < 0.1). Pre-specified potential adjustment variables included LAZ, WAZ, maternal education, marital status, HFIAS, SES index, health center, child sex, CRP, AGP, Hb, and anemia. Baseline and endline eThDP and EGRac were log-transformed to normality, but the change in eThDP and riboflavin concentrations from baseline to endline was normally distributed and thus not transformed. Log-transformed outcome variables were back-transformed (exponential) after regression. Analysis of covariance (ANCOVA) models were used to assess the impact of MNP on continuous outcomes (endline eThDP, EGRac, plasma folate, vitamin B_12_ concentrations), while a modified Poisson regression model [65] was used to assess the impact on dichotomous outcomes (prevalence of thiamine, riboflavin, folate, and B_12_ deficiencies).

Logistic regression models were used to examine potential risk factors associated with thiamine, riboflavin, folate, and B_12_ deficiencies at baseline, and analyses were adjusted for age at baseline, sex, and district of enrollment.

## Results

**Fig. 1:**
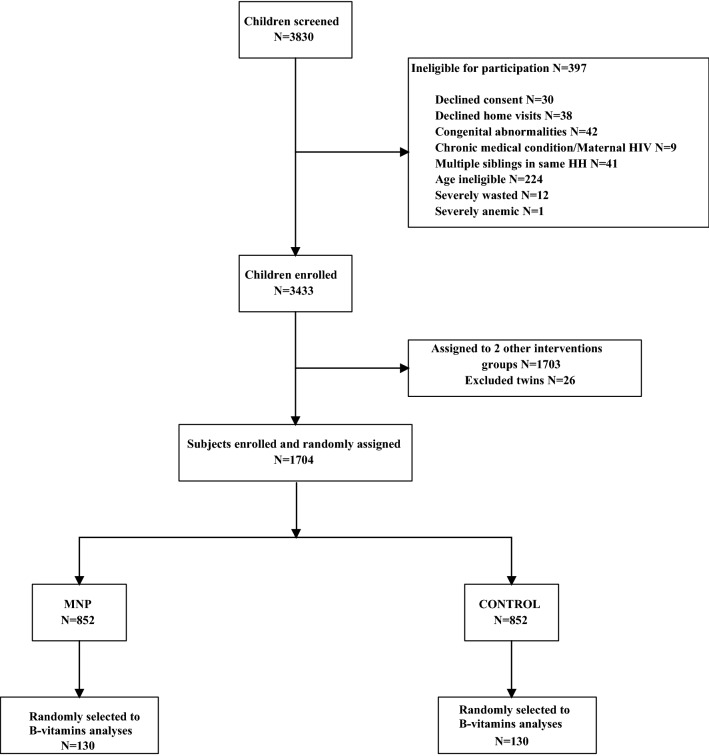
Lao Zinc Study flow diagram for B-vitamins samples collection. MNP, Micronutrient powder

Of the 3830 children screened for eligibility for the parent study, 3433 were enrolled and 3407 were eligible for analyses (26 twins excluded). A total of 1704 children were randomized to the MNP (*N* = 852) and the control (*N* = 852) groups. Among those, a random sub-sample of 260 children (*N* = 130 in each group) was selected for the present analyses (Fig. 1).

Mean age of the children was 13.1 ± 4.9 mo at enrollment (Table 1). At baseline, breastfeeding was high (84.1%) but reported IYCF practices showed that only 4.8% and 58.2% of children achieved MDD and MMF, respectively. The prevalence of stunting (36.5%) and underweight (31.2%) were both very high, and the prevalence of wasting was 12.3%. The prevalence of anemia was 64.6%, and about half of all children were deficient in thiamine (58.1%) and riboflavin (50.6%), yet deficiencies in folate and B_12_ were relatively low at 8.1% and 6.2% respectively. Mean maternal BMI was 21.3 ± 2.8 kg/m^2^ and 21.6% of the households reported severe or moderate food insecurity.

**Table 1:** Baseline characteristics of study participants by intervention group

Variables	All	MNP	Control
*N*	260	130	130
Age in months	13.1 ± 4.9	13.4 ± 4.9	12.8 ± 4.8
Females, *n* (%)	119 (45.8)	62 (47.7)	57 (43.9)
Breastfeeding, (%)	159 (84.1)	77 (83.7)	82 (84.5)
Minimum dietary diversity^a^, *n* (%)	9 (4.8)	5 (5.4)	4 (4.1)
Minimum meal frequency^b^, *n* (%)	110 (58.2)	50 (54.4)	60 (61.9)
Consumption of iron-rich foods, *n* (%)	122 (64.6)	61 (66.3)	61 (62.9)
Child anthropometric measures			
Length (cm)	71.6 ± 5.4	71.7 ± 5.6	71.4 ± 5.3
Weight (kg)	8.0 ± 1.2	8.0 ± 1.3	8.0 ± 1.2
MUAC (cm)	13.6 ± 1.0	13.6 ± 1.0	13.7 ± 0.9
LAZ	− 1.7 ± 1.0	− 1.7 ± 1.0	− 1.6 ± 0.9
WAZ	− 1.5 ± 0.9	− 1.6 ± 0.9	− 1.5 ± 0.8
WLZ	− 0.9 ± 0.9	− 0.9 ± 0.9	− 0.9 ± 0.9
Stunting, *n* (%)	95 (36.5)	54 (41.5)	41 (31.5)
Wasting, *n* (%)	32 (12.3)	17 (13.1)	15 (11.5)
Underweight, *n* (%)	81 (31.2)	48 (36.9)	33 (25.4)
CRP, mg/L	2.9 ± 6.2	2.6 ± 5.4	3.3 ± 7.0
Elevated CRP (> 5 mg/L), *n* (%)	40 (15.4)	19 (14.6)	21 (16.3)
AGP, g/L	0.8 ± 0.5	0.8 ± 0.5	0.8 ± 0.5
Elevated AGP (> 1 g/L), *n* (%)	58 (22.4)	32 (24.6)	26 (20.2)
Hemoglobin concentrations, g/L	105.5 ± 10.5	106.0 ± 11.3	105.0 ± 9.8
Anemia, *n* (%)	168 (64.6)	79 (60.8)	89 (68.5)
Thiamine concentrations, nmol/L	78.5 ± 57.2	78.4 ± 57.3	78.6 ± 57.2
Thiamine deficiency (< 70 nmol/L), *n* (%)	151 (58.1)	74 (56.9)	77 (59.2)
EGRac	1.44 ± 0.29	1.40 ± 0.24	1.48 ± 0.32
Riboflavin deficiency^c^ (EGRac > = 1.4), *n* (%)	130 (50.6)	58 (45.0)	72 (56.3)
Folate concentrations, nmol/L	22.1 ± 10.1	21.2 ± 9.6	23.0 ± 10.5
Folate deficiency (< 10 nmol/L), *n* (%)	21 (8.1)	11 (8.5)	10 (7.7)
Vitamin B_12_ concentrations, pmol/L	451.0 ± 180.8	466.0 ± 174.0	436.1 ± 187.0
Low or marginal vitamin B_12_ (< 221 pmol/L), *n* (%)	16 (6.2)	5 (3.9)	11 (8.5)
Maternal education (primary or lower), *n* (%)	171 (66.8)	91 (70.5)	80 (63.0)
Maternal BMI, kg/m^2^	21.3 ± 2.8	21.9 ± 3.0	20.8 ± 2.6
HFIAS, *n* (%)			
Food secure	98 (37.7)	54 (41.5)	44 (33.9)
Mildly food insecure access	106 (40.8)	46 (35.4)	60 (46.2)
Moderately food insecure access	34 (13.1)	17 (13.1)	17 (13.1)
Severely food insecure access	22 (8.5)	13 (10.0)	9 (6.9)

Values presented as mean ± standard deviation or *n* (%).

*AGP* α_1_-acid glycoprotein, *BMI* body mass index, *CRP* C-reactive protein, *EGRac* erythrocyte glutathione reductase activity coefficient, *HFIAS* household food insecurity access scale, *LAZ* length-for-age *z* score, *MNP* micronutrient powder, *MUAC* mid-upper arm circumference, *WAZ* weight-for-age *z *score, *WLZ* weight-for-length *z *score

^a^Proportion of children 6–23 months of age who receive foods from 4 or more food groups

^b^Proportion of breastfed and non-breastfed children 6–23 months of age who receive solid, semi-solid, or soft foods the minimum number of times or more

^c^*n* = 257 samples have been analyzed for riboflavin status

Over the course of the 9 month intervention, adherence calculated as the number of days the supplement was reportedly consumed divided by the number of days the child had access to the supplement, was 90%, and did not differ by intervention group (*p* = 0.375). In models adjusted for baseline thiamine concentrations, age, district, LAZ, WAZ, maternal education, marital status, HFIAS, SES index, and health center, MNP had no effect on endline thiamine concentration compared to the placebo and there was no difference in the prevalence of thiamine deficiency between groups (*p* = 0.924 and 0.881, respectively)(Table 2). Similarly, endline EGRac and prevalence of riboflavin deficiency did not differ significantly between the two groups (*p* = 0.184 and 0.364, respectively) in models adjusted for baseline EGRac, age, district WAZ, HFIAS, SES index, hemoglobin, and CRP. However, in models adjusted for baseline concentrations of folate, age, district, WAZ, HFIAS, SES index, Hb, and CRP, MNP significantly increased endline folate concentration compared to the placebo group (*p* < 0.001), resulting in a significantly lower prevalence of folate deficiency among children who received MNP compared to children who did not (1.6% vs. 17.4%, respectively; *p* = 0.015). Endline B_12_ concentrations and prevalence of B_12_ deficiency were similar between MNP and placebo groups (*p* = 0.963 and *p* = 0.491; respectively) in models adjusted for baseline concentrations of B_12_, age, district, anemia, and AGP.

**Table 2:** Effect of supplementation with daily micronutrient powder or daily placebo on endline thiamine, riboflavin, folate, and vitamin B_12_ status among young Laotian children

	MNP	Control	Effect size	*p* value
Thiamine, eThDP				
Endline thiamine concentration, nmol/L				
Minimally adjusted	109.5 ± 8.9	109.6 ± 8.9	− 0.1 ± 8.9	0.980
Fully adjusted^a^	110.6 ± 8.9	109.4 ± 8.9	1.2 ± 8.9	0.924
Prevalence of thiamine deficiency (< 70 nmol/L)				
Minimally adjusted	19.0	18.7	1.01	0.950
Fully adjusted^a^	18.1	18.8	0.96	0.881
Riboflavin, EGRac				
Endline EGRac				
Minimally adjusted	1.46 ± 0.3	1.48 ± 0.3	− 0.02 ± 0.3	0.314
Fully adjusted^b^	1.46 ± 0.3	1.49 ± 0.3	− 0.03 ± 0.3	0.184
Prevalence of riboflavin deficiency (EGRac > = 1.4), %				
Minimally adjusted	56.7	59. 9	0.95	0.541
Fully adjusted^b^	56.4	61.0	0.92	0.364
Folate				
Endline folate concentration, nmol/L				
Minimally adjusted	28.2 ± 0.8	19.8 ± 0.8	8.4 ± 0.9	< 0.001
Fully adjusted^c^	28.2 ± 0.8	19.9 ± 0.8	8.3 ± 0.9	< 0.001
Prevalence of folate deficiency (< 10 nmol/L), %				
Minimally adjusted	2.2	9.5	0.23	0.026
Fully adjusted^c^	1.6	17.4	0.09	0.015
Vitamin B_12_				
Endline vitamin B_12_ concentration, pmol/L				
Minimally adjusted	518.2 ± 24.5	521.0 ± 24.5	− 2.9 ± 24.6	0.874
Fully adjusted^d^	523.3 ± 24.6	515.9 ± 24.8	7.4 ± 24.6	0.678
Prevalence of vitamin B_12_ deficiency (< 221 pmol/L), %				
Minimally adjusted	4.4	1.4	3.14	0.381
Fully adjusted^d^	3.7	1.6	2.31	0.491

Results shown as geometric mean ± geometric SD for eThDP and EGRac (eThDP and EGRac were log-transformed, and then, the estimates were back-transformed using Excel’s exponential function) and arithmetic mean ± SD for folate and vitamin B_12_. ANCOVA regression models were used to examine the difference in mean eThDP, EGRac, folate, and vitamin B_12_ at endline. A modified Poisson regression model was used to estimate the prevalence ratio of thiamine, riboflavin, folate, and B12 deficiencies).

*EGRac* erythrocyte glutathione reductase activity coefficient; *eThDP* erythrocyte thiamine diphosphate, *Minimally adjusted* adjusted for baseline value, age, and district, *n* 257 samples have been analyzed for riboflavin status

^a^Fully adjusted = Minimally adjusted + LAZ, WAZ, maternal education, marital status, HFIAS, SES index, and health center

^b^Fully adjusted = Minimally adjusted + sex and CRP

^c^Fully adjusted = Minimally adjusted + WAZ, HFIAS, SES index, Hb, and CRP

^d^Fully adjusted = Minimally adjusted + anemia and AGP

Results from the associations between micronutrient deficiencies and potential risk factors at baseline show that children who were thiamine or riboflavin deficient were more likely to have lower WLZ at baseline. In addition, children who were riboflavin deficient were more likely to be wasted, and have lower weight and WLZ at baseline and children who were folate deficient were more likely to be stunted. There were no other associations between micronutrient deficiencies and growth outcomes (Table 3). Among IYCF indicators, thiamine and riboflavin deficiencies were positively associated with breastfeeding. Riboflavin and B_12_ deficiencies were negatively associated with Hb concentrations and riboflavin deficiency was positively associated with anemia. Elevated CRP was positively associated with riboflavin deficiency whereas there were no other associations between B-vitamins deficiencies and other markers of inflammation. Riboflavin deficiency was negatively associated with ferritin concentrations and positively associated with both sTfR concentrations and deficiency. Maternal characteristics were not associated with B-vitamin deficiencies, except for maternal education which was negatively associated with riboflavin deficiency. At a household level, riboflavin deficiency was negatively associated with SES index and positively associated with HFIAS, whereas no association was found with the other B-vitamins deficiencies.

**Table 3:** Associations between baseline thiamine, riboflavin, folate, and B_12_ deficiencies and potential risk factors among young Laotian children

	Thiamine (< 70 nmol/L)		EGRac (> = 1.4)		Folate (< 10 nmol/L)		B_12_ (< 221 pmol/L)	
	OR [95% CI]	*p*	OR [95% CI]	*p*	OR [95% CI]	*p*	OR [95% CI]	*p*
Child								
Weight, kg	0.89 [0.65–1.22]	0.488	**0.68 [0.49**–**0.93]**	**0.016**	0.85 [0.48–1.52]	0.589	0.72 [0.34–1.50]	0.377
Length, cm	1.10 [0.98–1.23]	0.099	0.94 [0.84–1.05]	0.259	0.86 [0.70–1.04]	0.126	0.95 [0.75–1.19]	0.651
LAZ	1.24 [0.94–1.65]	0.125	0.84 [0.64–1.10]	0.200	0.62 [0.37–1.04]	0.070	0.80 [0.45–1.41]	0.438
WAZ	0.91 [0.68–1.21]	0.528	**0.71 [0.53**–**0.95]**	**0.020**	0.84 [0.48–1.47]	0.536	0.72 [0.38–1.36]	0.311
WLZ	**0.71 [0.52**–**0.96]**	**0.024**	**0.69 [0.51**–**0.94]**	**0.017**	1.13 [0.65–1.94]	0.666	0.77 [0.41–1.44]	0.418
Stunting	0.83 [0.48–1.42]	0.494	1.31 [0.76–2.24]	0.334	**3.46 [1.29**–**9.29]**	**0.014**	1.11 [0.35–3.53]	0.864
Underweight	1.15 [0.65–2.02]	0.633	1.58 [0.90–2.78]	0.110	1.09 [0.40–2.94]	0.871	1.18 [0.33–4.21]	0.800
Wasting	2.17 [0.92–5.15]	0.078	**2.40 [1.03**–**5.62]**	**0.042**	0.32 [0.04–2.57]	0.286	0.66 [0.08–5.58]	0.706
Breastfeeding	**2.95 [1.14**–**7.63]**	**0.026**	**5.80 [2.13**–**15.81]**	**0.001**	0.56 [0.12–2.67]	0.467	-	-
Iron rich foods^a^	1.57 [0.81–3.07]	0.182	1.23 [0.62–2.43]	0.550	4.46 [0.53–37.10]	0.167	1.23 [0.31–4.91]	0.771
MDD^a^	0.56 [0.14–2.30]	0.423	0.33 [0.07–1.71]	0.188	1.92 [0.20–18.27]	0.571	3.48 [0.53–22.77]	0.193
MMF^a^	1.65 [0.87–3.14]	0.122	1.62 [0.85–3.10]	0.138	0.90 [0.23–3.51]	0.879	1.15 [0.31–4.24]	0.828
Hemoglobin, g/L	0.99 [0.96–1.01]	0.358	**0.94 [0.92**–**0.97]**	**0.000**	0.96 [0.92–1.00]	0.061	**0.94 [0.90**–**0.99]**	**0.019**
Anemia, %	1.42 [0.84–2.42]	0.193	**3.72 [2.11**–**6.59]**	**0.000**	2.25 [0.75–6.77]	0.149	1.42 [0.45–4.44]	0.551
CRP, mg/L	1.03 [0.87–1.23]	0.694	1.16 [0.98–1.39]	0.089	0.88 [0.64–1.23]	0.455	1.03 [0.72–1.48]	0.860
CRP > 5 mg/L	1.10 [0.53–2.25]	0.802	**2.32 [1.07**–**5.04]**	**0.033**	0.87 [0.23–3.30]	0.839	1.01 [0.21–5.00]	0.986
AGP, g/L	0.95 [0.59–1.53]	0.840	1.29 [0.80–2.10]	0.301	1.27 [0.53–3.07]	0.594	0.74 [0.28–1.97]	0.550
AGP > 1 g/L	0.69 [0.37–1.29]	0.242	1.02 [0.54–1.92]	0.960	1.37 [0.48–3.94]	0.559	0.23 [0.03–1.87]	0.168
RBP, µmol/L	0.94 [0.36–2.46]	0.893	0.64 [0.24–1.68]	0.362	0.82 [0.15–4.60]	0.819	0.09 [0.01–1.14]	0.063
Ferritin, µg/L	0.97 [0.72–1.30]	0.830	**0.72 [0.53**–**0.97]**	**0.031**	0.59 [0.34–1.04]	0.068	0.88 [0.49–1.59]	0.673
pF < 12 µg/L	1.14 [0.63–2.05]	0.662	1.76 [0.96–3.24]	0.069	2.34 [0.89–6.18]	0.085	1.82 [0.61–5.44]	0.285
sTfR, mg/L	0.70 [0.30–1.68]	0.424	**5.90 [2.17**–**16.09]**	**0.001**	1.73 [0.35–8.48]	0.502	1.57 [0.28–9.99]	0.610
sTfR > 8.3 mg/L	0.90 [0.53–1.52]	0.690	**2.37 [1.37**–**4.10]**	**0.002**	1.37 [0.49–3.78]	0.547	0.95 [0.33–2.78]	0.930
Maternal								
Age	0.99 [0.95–1.04]	0.713	1.02 [0.97–1.06]	0.437	1.02 [0.94–1.11]	0.611	1.00 [0.92–1.11]	0.853
Education	1.10 [0.63–1.94]	0.732	**0.46 [0.26**–**0.81]**	**0.007**	0.45 [0.14–1.48]	0.190	0.68 [0.20–2.25]	0.525
Marital status (couple)	1.19 [0.34–4.17]	0.783	3.62 [0.92–14.26]	0.065	0.57 [0.06–5.16]	0.618	0.78 [0.08–7.58]	0.833
BMI	1.00 [0.90–1.10]	0.938	0.98 [0.90–1.10]	0.743	0.93 [0.78–1.12]	0.451	1.00 [0.82–1.20]	0.962
Household								
SES index	0.93 [0.82–1.05]	0.253	**0.84 [0.73**–**0.96]**	**0.009**	0.80 [0.62–1.01]	0.064	1.03 [0.79–1.33]	0.836
HFIAS	1.00 [0.89–1.11]	0.972	**1.20 [1.06**–**1.36]**	**0.005**	1.06 [0.87–1.28]	0.573	0.89 [0.69–1.15]	0.376

Models adjusted for age at baseline, sex and district.

*MDD* Minimum dietary diversity, *MMF* Minimum meal frequency, *AGP* α_1_-acid glycoprotein, *BMI* body mass index; *CRP* C-reactive protein; *EGRac* erythrocyte glutathione reductase activity coefficient, *HFIAS* household food insecurity access scale, *LAZ* length-for-age *z* score; *MUAC* mid-upper arm circumference, *RBP* Retinol-Binding Protein, *SES* socio-economic status, *sTfR* soluble transferrin receptor, *WAZ* weight-for-age *z *score, *WLZ* weight-for-length *z *score.

^a^*n* = 188

## Discussion

This study indicates that the provision of a daily MNP containing 0.5 mg thiamine, 0.5 mg riboflavin, 150 μg folic acid, and 0.9 μg of vitamin B_12_ along with 11 other micronutrients for ~ 36 weeks improved folate, but did not change thiamine, riboflavin, and B_12_ status in young Laotian children. In addition, thiamine and riboflavin deficiencies were highly prevalent providing clear evidence of concurrent deficiencies of these micronutrients in this population. In contrast, both the prevalence of folate and B_12_ deficiencies were low. Moreover, deficiency in riboflavin was associated with a number of concurrent factors such as child weight, WAZ, WLZ, wasting, breastfeeding status Hb concentrations and anemia, elevated CRP, ferritin, sTfR, maternal education, household SES index, and HFIAS, whereas thiamine deficiency was associated with WLZ and breastfeeding status and folate and B_12_ deficiencies were associated with stunting and hemoglobin concentrations, respectively.

There is a lack of evidence of the impact of MNP supplementation on B-vitamin status in young children, making any comparisons with the current literature difficult. A pooled analysis of the four country IRIS trials reported that a daily MMN supplementation of infants 6–11 months of age significantly improved riboflavin status compared with a placebo [39]. In a 6 month trial among Ugandan children under 5 years old, MMN supplementation increased folate and B_12_ concentrations [41]. A 12 month LNS supplementation trial among Honduran children aged 6–18 mo showed an improvement in folate and B_12_ status [42] and a daily supplementation of a high-B_12_ LNS for 1 year among marginally stunted Nepalese infants aged 6–11 months, substantially increased cobalamin concentration in the intervention group compared to the children who were given LNS without cobalamin [66]. Moreover, a 2 year LNS supplementation for 6–24 mo children recruited from birth, resulted in a significantly lower prevalence of B_12_ deficiency in Kenya and Bangladesh and a lower prevalence of folate deficiency in Kenya [67]. Additional studies examining the effects of MNP on B-vitamins status are needed to better understand whether MNP is an effective intervention for improving the status of B-vitamins in young children in LMIC contexts.

Although this trial improved folate status, it failed to show any benefits of MNP supplementation on thiamine, riboflavin, and B_12_ status. The lack of impact of MNP on thiamine and riboflavin was surprising given the high prevalence of the deficiencies of these two B-vitamins; in addition, the absence of impact on B_12_ was also difficult to interpret given that the intervention had an impact on folate. We are uncertain as to why this was the case, but there are several possible explanations. First, plasma folate concentration is an indicator reflecting recent dietary folate intake, which is highly responsive to interventions with folic acid [68]. Second, as previously reported in the parent trial, MNP improved iron and zinc status, but did not have any impact on retinol-binding protein, an indicator of vitamin A status [69], suggesting that MNP supplementation impacted only select indicators of micronutrient status in young Laotian children. Third, although the MNP contained standard doses of thiamine, riboflavin, and B_12_, it may be that the doses of thiamine, riboflavin, and B_12_ in the MNP were insufficient to impact biomarker status or that absorption and bioavailability of thiamine, riboflavin, and B_12_ in the MNP were too low to improve the status of these vitamins in a highly deficient population. However, these are the same doses that were used in the previous MMN interventions trials in Peru, South Africa, Indonesia, and Vietnam [70]. Fourth, the MNP were produced by DSM Fortitech and vitamin content was verified as part of the producer’s standard quality assurance. Unfortunately, we did not test the content of these B-vitamins in an independent laboratory and thus cannot be certain whether the vitamin content remained stable throughout the course of the study. Finally, it is unlikely that the duration of our study (9 months) was a significant factor limiting the efficacy of MNP supplementation on thiamine, riboflavin, and B_12_ status, given that it was of longer duration than the previously cited MMN trials which showed a significant impact [39]. It is also unlikely that adherence to supplementation was a significant factor limiting the efficacy of the intervention given that reported consumption was 90% over the course of the intervention.

Evidence regarding potential risk factors associated with the deficiencies of thiamine, riboflavin, folate, and B_12_ status in young children in LMIC contexts is lacking. In the present study, riboflavin deficiency was the B-vitamin most often associated with concurrent risk factors. These factors tended to cluster into three main categories: indicators of growth, indicators of iron status, and household wealth (SES and HFIAS). These findings are in accordance with previous studies which reported that riboflavin deficiency may influence iron metabolism [25]. Additional research is needed regarding the risk factors associated with deficiencies of B-vitamins in young children.

In this study, thiamine status was determined in RBC and eThDP concentrations were substantially lower than what has been reported among Cambodian children of the same age using the same laboratory assay [71]. Given that there was no universally accepted cut-off value for thiamine deficiency, we used a cut-off of < 70 nmol/L reported in the 1998 Institute of Medicine DRI chapter [51]. In sensitivity analyses using cut-offs values of < 120 and < 180 nmol/L reported by the previous studies, we found higher prevalences of thiamine deficiency in our study population but no impact on the prevalence of thiamine deficiency using the aforementioned cut-offs. Similarly, plasma folate concentrations were lower than what has been reported in children from other low-income countries [31, 32, 72]. In contrast, in the present study, plasma B_12_ concentrations were higher and riboflavin biomarker status was much worse compared to what has been reported in the previous studies [31, 32, 39, 41]. There is a need to assess B-vitamins in nationally representative surveys to better understand the extent of these deficiencies in vulnerable population groups.

Notable strengths of this study are its randomized placebo-controlled double-blind design, its rigorous data collection, and its implementation in a setting with concurrent micronutrient deficiencies where participants should have had the potential to respond to the provided interventions. The lack of universally accepted cut-off value for thiamine deficiency resulted in a wide range in the prevalence of thiamine deficiency across studies [13, 72] making it difficult to determine the severity of thiamine deficiency as a public health concern. In the present study, we used a cutoff of eThDP < 70 nmol/L and found that 58% of children were deficient in thiamine. This prevalence was higher than the prevalence of 13–30% reported by two studies in Lao PDR in which thiamine deficiency was defined both in terms of a basal erythrocyte transketolase (ETK) activity < 0.59 micromoles/min/gHb and an activation coefficient of ETK α > 31% [14, 19, 20, 65]. However, established cut-offs exist for folate, riboflavin, and B_12_ deficiencies, and prevalence of folate, riboflavin, and B_12_ deficiencies in the present study were comparable, higher, or lower than in the previous studies [29–32, 39].

## Conclusion

In the present study, the provision of a daily MNP for 9 months increased only folate but not thiamine, riboflavin, or B_12_ status in young Laotian children, suggesting that MNP at the administered doses only impacted select indicators of micronutrient status in this population.

## References

[1] Victora CG, Christian P, Vidaletti LP (2021). Revisiting maternal and child undernutrition in low-income and middle-income countries: variable progress towards an unfinished agenda. Lancet.

[2] Brown KH, Moore SE, Hess SY (2021). Increasing the availability and utilization of reliable data on population micronutrient (MN) status globally: the MN data generation initiative. Am J Clin Nutr.

[3] Mark HE, Houghton LA, Gibson RS (2016). Estimating dietary micronutrient supply and the prevalence of inadequate intakes from national food balance sheets in the South Asia regiona. Asia Pac J Clin Nutr.

[4] McNulty H, Scott JM (2008). Intake and status of folate and related B-vitamins: considerations and challenges in achieving optimal status. Br J Nutr.

[5] Johnson CR, Fischer PR, Thacher TD (2019). Thiamin deficiency in low- and middle-income countries: disorders, prevalences, previous interventions and current recommendations. Nutr Health.

[6] Wieringa FT, Dijkhuizen MA, Berger J (2019). Micronutrient deficiencies and their public health implications for South-East Asia. Curr Opin Clin Nutr Metab Care.

[7] Kraemer K, Semba RD, Eggersdorfer M, Schaumberg DA (2012). Introduction: the diverse and essential biological functions of vitamins. Ann Nutr Metab.

[8] Manzetti S, Zhang J, van der Spoel D (2014). Thiamin function, metabolism, uptake, and transport. Biochemistry.

[9] Thakur K, Tomar SK, Singh AK (2017). Riboflavin and health: a review of recent human research. Crit Rev Food Sci Nutr.

[10] Troen AM (2012). Folate and vitamin B_12_: function and importance in cognitive development. Nestle Nutr Inst Workshop Ser.

[11] Luxemburger C, White NJ, ter Kuile F (2003). Beri-beri: the major cause of infant mortality in Karen refugees. Trans R Soc Trop Med Hyg.

[12] Fattal-Valevski A, Azouri-Fattal I, Greenstein YJ (2009). Delayed language development due to infantile thiamine deficiency. Dev Med Child Neurol.

[13] Whitfield KC, Bourassa MW, Adamolekun B (2018). Thiamine deficiency disorders: diagnosis, prevalence, and a roadmap for global control programs. Ann N Y Acad Sci.

[14] Smith TJ, Johnson CR, Koshy R (2021). Thiamine deficiency disorders: a clinical perspective. Ann N Y Acad Sci.

[15] Swaminathan S, Thomas T, Kurpad AV (2015). B-vitamin interventions for women and children in low-income populations. Curr Opin Clin Nutr Metab Care.

[16] Allen LH (2008). Causes of vitamin B_12_ and folate deficiency. Food Nutr Bull.

[17] Strand TA, Taneja S, Kumar T (2015). Vitamin B_12_, folic acid, and growth in 6- to 30 month-old children: a randomized controlled trial. Pediatrics.

[18] Molloy AM, Kirke PN, Troendle JF (2009). Maternal vitamin B_12_ status and risk of neural tube defects in a population with high neural tube defect prevalence and no folic acid fortification. Pediatrics.

[19] Lao Statistics Bureau. 2018. Lao social indicator survey II 2017, survey findings report. Vientiane, Lao PDR: Lao statistics bureau and UNICEF.

[20] Smith TJ, Hess SY (2021). Infantile thiamine deficiency in South and Southeast Asia: an age-old problem needing new solutions. Nutr Bull.

[21] Khounnorath S, Chamberlain K, Taylor AM (2011). Clinically unapparent infantile thiamin deficiency in Vientiane. Laos PLoS Negl Trop Dis.

[22] Barennes H, Sengkhamyong K, René JP, Phimmasane M (2015). Beriberi (thiamine deficiency) and high infant mortality in northern Laos. PLoS Negl Trop Dis.

[23] Hoey L, McNulty H, Strain JJ (2009). Studies of biomarker responses to intervention with riboflavin: a systematic review. Am J Clin Nutr.

[24] Abrams SA, Mushi A, Hilmers DC (2003). A multinutrient-fortified beverage enhances the nutritional status of children in Botswana. J Nutr.

[25] Rohner F, Zimmermann MB, Wegmueller R (2007). Mild riboflavin deficiency is highly prevalent in school-age children but does not increase risk for anaemia in Côte d’Ivoire. Br J Nutr.

[26] Steyn N, Eksteen G, Senekal M (2016). Assessment of the dietary Intake of school children in South Africa: 15 Years after the first national study. Nutrients.

[27] López de Romaña G, Cusirramos S, López de Romaña D, Gross R (2005). Efficacy of multiple micronutrient supplementation for improving anemia, micronutrient status, growth, and morbidity of Peruvian infants. J Nutr.

[28] Smuts CM, Dhansay MA, Faber M (2005). Efficacy of multiple micronutrient supplementation for improving anemia, micronutrient status, and growth in South African infants. J Nutr.

[29] Taneja S, Bhandari N, Strand TA (2007). Cobalamin and folate status in infants and young children in a low-to-middle income community in India. Am J Clin Nutr.

[30] Ng’eno BN, Perrine CG, Whitehead RD, (2017). High prevalence of vitamin B_12_ deficiency and no folate deficiency in young children in Nepal. Nutrients.

[31] Ulak M, Chandyo RK, Adhikari RK (2014). Cobalamin and folate status in 6 to 35 months old children presenting with acute diarrhea in Bhaktapur. Nepal PLoS One.

[32] Ulak M, Chandyo RK, Thorne-Lyman AL (2016). Vitamin status among breastfed infants in Bhaktapur. Nepal Nutrients.

[33] Muthayya S, Rah JH, Sugimoto JD (2013). The global hidden hunger indices and maps: an advocacy tool for action. PLoS ONE.

[34] Whitfield KC, Karakochuk CD, Kroeun H (2016). Perinatal consumption of thiamine-fortified fish sauce in rural Cambodia: a randomized clinical trial. JAMA Pediatr.

[35] Lassi ZS, Mansoor T, Salam RA (2014). Essential pre-pregnancy and pregnancy interventions for improved maternal, newborn and child health. Reprod Health.

[36] Duggan C, Srinivasan K, Thomas T (2014). Vitamin B_12_ supplementation during pregnancy and early lactation increases maternal, breast milk, and infant measures of vitamin B_12_ status. J Nutr.

[37] Ziaei S, Rahman A, Raqib R (2016). A prenatal multiple micronutrient supplement produces higher maternal vitamin B_12_ concentrations and similar folate, ferritin, and zinc concentrations as the standard 60 mg iron plus 400 μg folic acid supplement in rural Bangladeshi women. J Nutr.

[38] Hop LT, Berger J (2005). Multiple micronutrient supplementation improves anemia, micronutrient nutrient status, and growth of Vietnamese infants: double-blind, randomized, placebo-controlled trial. J Nutr.

[39] Smuts CM, Lombard CJ, Benadé AJS (2005). Efficacy of a foodlet-based multiple micronutrient supplement for preventing growth faltering, anemia, and micronutrient deficiency of infants: the four country IRIS trial pooled data analysis. J Nutr.

[40] Untoro J, Karyadi E, Wibowo L (2005). Multiple micronutrient supplements improve micronutrient status and anemia but not growth and morbidity of Indonesian infants: a randomized, double-blind, placebo-controlled trial. J Nutr.

[41] Ndeezi G, Tumwine JK, Ndugwa CM (2011). Multiple micronutrient supplementation improves vitamin B_12_ and folate concentrations of HIV infected children in Uganda: a randomized controlled trial. Nutr J.

[42] Siega-Riz AM, Estrada Del Campo Y, Kinlaw A (2014). Effect of supplementation with a lipid-based nutrient supplement on the micronutrient status of children aged 6–18 months living in the rural region of Intibucá, Honduras. Paediatr Perinat Epidemiol.

[43] Salam RA, MacPhail C, Das JK, Bhutta ZA (2013). Effectiveness of micronutrient powders (MNP) in women and children. BMC Public Health.

[44] De-Regil LM, Suchdev PS, Vist GE (2011). Home fortification of foods with multiple micronutrient powders for health and nutrition in children under two years of age. Cochrane Database Syst Rev.

[45] Wessells KR, Brown KH, Kounnavong S (2018). Comparison of two forms of daily preventive zinc supplementation versus therapeutic zinc supplementation for diarrhea on young children’s physical growth and risk of infection: study design and rationale for a randomized controlled trial. BMC Nutr.

[46] WHO Multicentre Growth Reference Study Group (2006). WHO child growth standards based on length/height, weight and age. Acta Paediatr Suppl.

[47] European Union, Ministry of health, MMG, UNICEF (2011) What is super kid? Counseling cards. Ministry of health. Vientiane, Lao PDR

[48] Cogill B (2003) Anthropometric indicators measurement guide. Food and nutrition technical assistance project, academy for educational development, Washington, DC

[49] Hampel D, Shahab-Ferdows S, Adair LS (2016). Thiamin and riboflavin in human milk: effects of lipid-based nutrient supplementation and stage of lactation on vitamer secretion and contributions to total vitamin content. PLoS ONE.

[50] Erhardt JG, Estes JE, Pfeiffer CM (2004). Combined measurement of ferritin, soluble transferrin receptor, retinol binding protein, and C-reactive protein by an inexpensive, sensitive, and simple sandwich enzyme-linked immunosorbent assay technique. J Nutr.

[51] Institute of Medicine (US) (1998) Standing committee on the scientific evaluation of dietary reference intakes and its panel on folate, other B vitamins, and choline. Dietary Reference Intakes for Thiamin, Riboflavin, Niacin, Vitamin B6, Folate, Vitamin B12, Pantothenic Acid, Biotin, and Choline. National Academies Press, Washington, DC 23193625

[52] Selhub J, Jacques PF, Dallal G (2008). The use of blood concentrations of vitamins and their respective functional indicators to define folate and vitamin B_12_ status. Food Nutr Bull.

[53] Rajan S, Wallace JI, Beresford SAA (2002). Screening for cobalamin deficiency in geriatric outpatients: prevalence and influence of synthetic cobalamin intake. J Am Geriatr Soc.

[54] World Health Organization (2007) WHO child growth standards: head circumference-for-age, arm circumference-for-age, triceps skinfold-for-age and subscapular skinfold-for-age: methods and development. World health organization. https://apps.who.int/iris/handle/10665/43706

[55] Thurnham DI, McCabe LD, Haldar S (2010). Adjusting plasma ferritin concentrations to remove the effects of subclinical inflammation in the assessment of iron deficiency: a meta-analysis. Am J Clin Nutr.

[56] World Health Organization (2020). WHO guideline on use of ferritin concentrations to assess iron status in individuals and populations.

[57] Phiri KS, Calis JCJ, Siyasiya A (2009). New cut-off values for ferritin and soluble transferrin receptor for the assessment of iron deficiency in children in a high infection pressure area. J Clin Pathol.

[58] Brown KH, Peerson JM, Baker SK, Hess SY (2009). Preventive zinc supplementation among infants, preschoolers, and older prepubertal children. Food Nutr Bull.

[59] Jaeggi T, Kortman GAM, Moretti D (2015). Iron fortification adversely affects the gut microbiome, increases pathogen abundance and induces intestinal inflammation in Kenyan infants. Gut.

[60] Adu-Afarwuah S, Lartey A, Brown KH (2008). Home fortification of complementary foods with micronutrient supplements is well accepted and has positive effects on infant iron status in Ghana. Am J Clin Nutr.

[61] Hess SY, Barffour MA, Hinnouho GM (2018) Lao Zinc Study. Open Science Framework. https://osf.io/5bq9c

[62] Vyas S, Kumaranayake L (2006). Constructing socio-economic status indices: how to use principal components analysis. Health Policy Plan.

[63] Coates J, Anne S, Paula B (2007) Household Food Insecurity Access Scale (HFIAS) for Measurement of Household Food Access: Indicator Guide (v. 3). Washington, DC: FHI 360/FANTA

[64] World Health Organization (2010) Indicators for assessing infant and young child feeding practices. Part II: measurement. World health organization, Geneva

[65] Zou G (2004). A modified poisson regression approach to prospective studies with binary data. Am J Epidemiol.

[66] Strand TA, Ulak M, Hysing M (2020). Effects of vitamin B_12_ supplementation on neurodevelopment and growth in Nepalese infants: a randomized controlled trial. PLoS Med.

[67] Stewart CP, Dewey KG, Lin A (2019). Effects of lipid-based nutrient supplements and infant and young child feeding counseling with or without improved water, sanitation, and hygiene (WASH) on anemia and micronutrient status: results from 2 cluster-randomized trials in Kenya and Bangladesh. Am J Clin Nutr.

[68] Bailey LB, Stover PJ, McNulty H (2015). Biomarkers of nutrition for development-folate review. J Nutr.

[69] Barffour MA, Hinnouho G-M, Kounnavong S (2019). Effects of daily zinc, daily multiple micronutrient powder, or therapeutic zinc supplementation for diarrhea prevention on physical growth, anemia, and micronutrient status in rural Laotian children: a randomized controlled trial. J Pediatr.

[70] Lock G (2003). The foodLET vehicle designed for and used in the IRIS I intervention. Food Nutr Bull.

[71] Whitfield KC, Smith G, Chamnan C (2017). High prevalence of thiamine (vitamin B_1_) deficiency in early childhood among a nationally representative sample of Cambodian women of childbearing age and their children. PLoS Negl Trop Dis.

[72] Eneroth H, El Arifeen S, Persson L-A (2010). Maternal multiple micronutrient supplementation has limited impact on micronutrient status of Bangladeshi infants compared with standard iron and folic acid supplementation. J Nutr.

